# Harnessing Regulatory T Cells to Modulate Acute Brain Injury: From Mechanisms to Therapy

**DOI:** 10.1007/s12035-026-06011-7

**Published:** 2026-06-29

**Authors:** Junaid Ansari, Sarvin Sasannia, Mykola Matsyuk, Christopher Jackson, Paul Nyquist

**Affiliations:** 1 Neurocritical Care Division, Department of Anesthesiology and Critical Care Medicine, The Johns Hopkins Hospital, Baltimore, MD USA; 2 Department of Neurology, Johns Hopkins University School of Medicine, Baltimore, MD USA; 3 F.M. Kirby Research Center for Functional Brain Imaging, Kennedy Krieger Research Institute, Baltimore, MD USA; 4 Department of Neurosurgery, The Johns Hopkins Hospital, Baltimore, MD USA; 5 Department of Neurology, LSU Health Shreveport, Shreveport, LA USA

**Keywords:** T lymphocytes, Neuroinflammation, Regulatory T cells, Blood–brain barrier, Stroke, Traumatic brain injury, Cerebral aneurysms

## Abstract

Neuroinflammation is a defining feature of acute brain injury such as acute ischemic stroke, traumatic brain injury, intracerebral hemorrhage, and subarachnoid hemorrhage, driven by complex immune interactions within the central nervous system (CNS). Among these, regulatory T cells (Tregs) have emerged as pivotal modulators that counteract the deleterious effects of effector T cell subsets, including Th1 and Th17 cells, which exacerbate blood–brain barrier (BBB) disruption, leukocyte infiltration, and glial activation. In contrast, Tregs exert immunosuppressive and tissue-reparative effects that promote neuroprotection, BBB integrity, and resolution of inflammation. This review examines the mechanistic roles of Tregs in modulating CNS-resident cells such as microglia, astrocytes, and endothelial cells, with emphasis on their capacity to suppress inflammation and support functional recovery. We also highlight emerging therapeutic strategies including low-dose interleukin-2 for selective Treg expansion, exosome-based immunomodulation, and chemokine-directed cell trafficking as promising avenues for clinical intervention. Finally, we discuss translational efforts to harness Tregs in acute neuroinflammatory disease, highlighting their potential as key targets for future therapies.

**Supplementary Information:**

The online version contains supplementary material available at 10.1007/s12035-026-06011-7.

## Introduction

Neuroinflammation, defined by immune activation within the central nervous system (CNS), is a hallmark of numerous acute brain injury disorders, including acute ischemic stroke (AIS), intracerebral hemorrhage (ICH), aneurysmal subarachnoid hemorrhage (aSAH), and traumatic brain injury (TBI) [1–3].

Among the key regulators of neuroinflammation are T lymphocytes, which serve not only in pathogen defense but also in shaping immune resolution and tissue repair [4, 5]. CD4⁺ T cells differentiate into functionally distinct subsets: Th1, Th2, and Th17 cells as well as regulatory T cells (Tregs). Th1 and Th17 cells drive proinflammatory responses, whereas Th2 and Tregs are generally associated with anti-inflammatory and reparative functions [6–9]. Th1 cells produce interferon-gamma (IFN-γ), which increases BBB permeability, while Th17 cells release interleukin-17 (IL-17), promoting neutrophil recruitment and amplifying cytokine cascades [10–12]. In contrast, Th2 cells secrete cytokines such as IL-4 and IL-13, which can modulate microglial activation and support tissue repair in a context-dependent manner. Tregs further limit neuroinflammation through the secretion of anti-inflammatory cytokines such as IL-10 and transforming growth factor-beta (TGF-β), stabilization of the BBB, and modulation of resident glial responses via direct cell-to-cell interactions and the inhibition of proinflammatory signaling pathways like STAT3 and NF-κB [13–16]. The delicate balance between proinflammatory and regulatory T cell subsets is a major determinant of CNS injury progression and repair [17].

Over the past decade, several foundational studies have demonstrated that Tregs exert neuroprotective effects across major forms of acute brain injury [13, 18–20]. In AIS, Tregs reduce infarct progression by limiting microglial activation and maintaining BBB integrity [13, 21, 22]. In ICH, regulatory T cells attenuate perihematomal inflammation and promote polarization of macrophages and microglia toward reparative M2 phenotypes [23, 24]. In aSAH, impaired Treg responses and altered Th/Treg balance are associated with enhanced neuroinflammation and may contribute to secondary complications such as delayed cerebral ischemia [20, 25]. In TBI, Treg depletion exacerbates neuroinflammation, increasing T cell infiltration, glial activation, and proinflammatory cytokine signaling, which contributes to worsened neurological outcomes [18, 26].

Despite these advances, critical gaps remain, including incomplete characterization of Treg–innate immune interactions, differences in Treg phenotypes across CNS compartments, and limited translational success of Treg-directed therapies such as IL-2 expansion or adoptive transfer [27–30]. This review builds on prior work by integrating mechanistic and disease-specific insights across AIS, ICH, aSAH, and TBI, highlighting shared and distinct Treg pathways, and outlining therapeutic strategies that target Treg recruitment, expansion, and cytokine signaling to modulate acute neuroinflammation [31, 32].

**Fig. 1:**
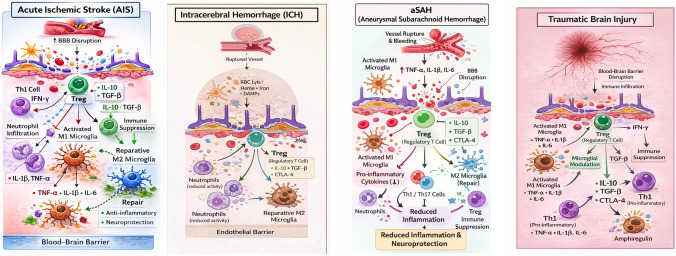
Immunopathological mechanisms of T cell subsets in acute ischemic stroke, intracerebral hemorrhage, cerebral aneurysm, and traumatic brain injury. In AIS, cerebral ischemia leads to blood–brain barrier (BBB) disruption and recruitment of peripheral immune cells, including neutrophils and T lymphocytes, into the injured brain parenchyma. Activated Th1 cells produce interferon-γ (IFN-γ) and amplify inflammatory signaling, contributing to microglial activation and secondary neuronal injury. In contrast, Tregs suppress inflammatory responses through IL-10 and TGF-β signaling and help preserve BBB integrity, thereby limiting infarct progression and neuroinflammation [13, 32, 33]

Figure 1 illustrates the immunopathological processes across major acute CNS disorders, emphasizing the contribution of T lymphocytes to BBB disruption, cytokine signaling, and immune cell recruitment in AIS, ICH, cerebral aneurysm, and TBI.

In ICH, rupture of cerebral vessels causes blood extravasation and release of erythrocyte-derived damage-associated molecular patterns (DAMPs) such as heme and iron, which activate innate immune responses and promote neutrophil and macrophage infiltration. Experimental studies demonstrate that Tregs attenuate perihematomal inflammation and promote reparative M2 microglial/macrophage polarization through IL-10 and TGF-β-dependent pathways, facilitating hematoma resolution and limiting secondary brain injury [23, 24, 34].

In aSAH, aneurysmal rupture results in subarachnoid bleeding and intense neurovascular inflammation, characterized by microglial activation and production of proinflammatory cytokines such as TNF-α, IL-1β, and IL-6. Tregs suppress these inflammatory responses by releasing IL-10 and TGF-β and inhibiting effector T cell activation, thereby mitigating vascular inflammation and reducing the risk of delayed cerebral ischemia [20, 25, 35].

In TBI, mechanical injury disrupts the BBB and triggers infiltration of peripheral immune cells and activation of resident microglia. Th1-mediated immune responses and IFN-γ signaling contribute to sustained neuroinflammation, whereas Tregs suppress effector T cell responses and regulate microglial activation via IL-10, TGF-β, CTLA-4, and amphiregulin, promoting resolution of inflammation and tissue repair. Experimental depletion of Tregs exacerbates neuroinflammation and worsens neurological outcomes after TBI [17, 18, 27]. Image created using biorender.com.

## CNS Immune Compartmentalization and Treg Trafficking

The CNS maintains a specialized form of immune compartmentalization that tightly regulates the trafficking, activation, and function of T lymphocytes, including Tregs, during injury and repair [36]. This immune specialization plays a defining role in acute CNS disorders [37]. At the core of this compartmentalization is the BBB, a selectively permeable interface formed by brain microvascular endothelial cells (BMECs), pericytes, astrocytes, and the basement membrane [36, 38, 39]. Under physiological conditions, the BBB restricts paracellular movement of immune cells and maintains CNS homeostasis [40]. However, in the context of ABI, BBB disruption enables the entry of peripheral immune cells including proinflammatory Th1 and Th17 cells, as well as Tregs into the parenchyma [41]. Importantly, Tregs modulate BBB integrity by downregulating endothelial adhesion molecules such as ICAM-1 and VCAM-1 and secreting anti-inflammatory cytokines like IL-10 and TGF-β, which help restore barrier function and prevent excessive immune infiltration [13, 21].

Within the neurovascular unit, Tregs interact with astrocytes and microglia, influencing the inflammatory milieu following acute brain injury [42, 43]. Experimental studies in AIS and other CNS injury models suggest that Tregs can modulate glial activation and promote anti-inflammatory or reparative phenotypes within the injured brain [13, 44]. Chemokine signaling within the neurovascular unit, including astrocyte-derived mediators that regulate leukocyte trafficking across the BBB, plays an important role in coordinating immune cell recruitment to sites of injury [45, 46].

In this context, Tregs have been shown to attenuate microglial production of proinflammatory cytokines such as IL-1β and TNF-α, thereby limiting secondary neuroinflammation and downstream leukocyte recruitment [42, 47]. Specifically, Tregs utilize mediators like interleukin-10 (IL-10) and amphiregulin (AREG) to suppress neurotoxic astrogliosis and promote a shift toward a neuroprotective microglial state [13, 44]. Together, these interactions highlight the role of Tregs as modulators of glial responses within the injured CNS microenvironment [48].

Beyond the BBB, CNS immune regulation is also influenced by glymphatic and meningeal lymphatic drainage pathways, which facilitate clearance of inflammatory mediators and trafficking of CNS-derived antigens toward cervical lymph nodes [49–51]. Impairment of these systems has been observed in AIS and TBI and may contribute to persistent neuroinflammation [49, 52]. Emerging evidence suggests that Treg-mediated suppression of astrocytic and inflammatory signaling pathways may indirectly influence glymphatic function, although direct mechanistic links remain incompletely understood [18, 27].

## Mechanisms of Tregs in Acute Brain Injury

### Temporal Dynamics and Phenotypic Characterization of CNS T Cells

T lymphocytes are critical regulators of immune responses in the CNS, contributing to both injury propagation and repair following acute and chronic neuroinflammatory insults [53]. While much of the foundational research has focused on chronic autoimmune diseases like multiple sclerosis (MS) where autoreactive CD4⁺ and CD8⁺ T cells target myelin and exacerbate demyelination [54], more recent work has emphasized their involvement in acute brain injury [53, 55, 56]. Following ABI, Treg dynamics unfold in distinct temporal phases that reflect their evolving immunological roles.

1. Early phase (0–24 h): In the immediate aftermath of injury, systemic immune responses are characterized by rapid activation of innate immunity and redistribution of peripheral lymphocyte populations, including splenic contraction and early lymphopenia [57]. While effector T cell subsets, including Th1 and Th17 cells, contribute to early neuroinflammatory signaling, Tregs demonstrate limited early accumulation within the CNS. During this phase, Tregs are thought to primarily modulate systemic immune responses rather than exert direct effects within the injured brain [13, 22, 58].
2. Intermediate phase (24–72 h): As BBB permeability increases following injury, Tregs begin to traffic into the injured CNS, with their accumulation becoming more apparent within the first 24–72 h. During this phase, Tregs emerge as critical modulators of neuroinflammation, counterbalancing the peak influx of proinflammatory effector T cells. Mechanistically, Tregs exert immunosuppressive effects through the secretion of anti-inflammatory cytokines, including IL-10 and TGF-β, and by attenuating microglial and astrocytic activation [18, 27, 59, 60].
3. Subacute phase (3–7 days): This phase represents the peak of Treg activity within the injured CNS. Expanded Treg populations promote resolution of neuroinflammation by modulating innate immune responses, including shifting microglia and infiltrating macrophages toward a reparative (M2-like) phenotype. In parallel, Tregs suppress endothelial activation and contribute to restoration of BBB integrity through stabilization of tight junction proteins such as claudin-5, occludin, and ZO-1. These effects collectively reduce secondary neuronal injury and coincide with a decline in proinflammatory Th1/Th17 signaling, marking a critical window for limiting delayed neurotoxicity [13, 18, 21, 27, 42, 58, 60].
4. Late phase (> 7 days): Tregs adopt a tissue-resident phenotype characterized by enhanced FOXP3 expression, IL-10 secretion, and local clonal expansion to CNS antigens. In this chronic–resolution stage, Tregs promote angiogenesis, remyelination, synaptic remodeling, and long-term neurorepair. Persistent Treg dysfunction in this phase has been linked to cognitive decline after stroke and prolonged neuroinflammation in TBI and aSAH [61–63].

Together, these temporally defined roles illustrate that Tregs do not simply suppress inflammation but rather evolve from peripheral immune regulators to central orchestrators of CNS repair. Incorporating temporal kinetics provides a more accurate understanding of how Tregs influence each stage of acute brain injury pathology [13, 58, 63].

CNS recruitment of T cells is mediated by endothelial activation at the blood–brain barrier, including upregulation of adhesion molecules such as ICAM-1 and VCAM-1 and chemokine signaling that facilitates lymphocyte adhesion and transmigration [40, 64].

Once within the CNS, T cells exhibit functional polarization. Th1 cells, through interferon-gamma (IFN-γ), promote microglial activation, while Th17 cells, via interleukin-17 (IL-17), contribute to blood–brain barrier disruption and neutrophil recruitment [65]. These proinflammatory subsets predominate in the acute phase of acute brain injury and are associated with worse outcomes [4, 66].

In contrast, regulatory T cells (Tregs) exert key immunomodulatory effects within the inflamed CNS. Beyond their direct suppressive functions, Tregs modulate CD4⁺ T cell polarization by inhibiting Th1 and Th17 responses and, in certain contexts, promoting a shift toward Th2-associated phenotypes, thereby fostering an anti-inflammatory and reparative environment [67]. Through the secretion of interleukin-10 (IL-10), transforming growth factor-beta (TGF-β), and amphiregulin (AREG), Tregs attenuate effector T cell activation, promote alternative microglial polarization, and support neurovascular repair [4, 13, 68]. Tregs also reinforce blood–brain barrier integrity by enhancing tight junction protein expression and reducing endothelial activation [13, 21].

**Fig. 2:**
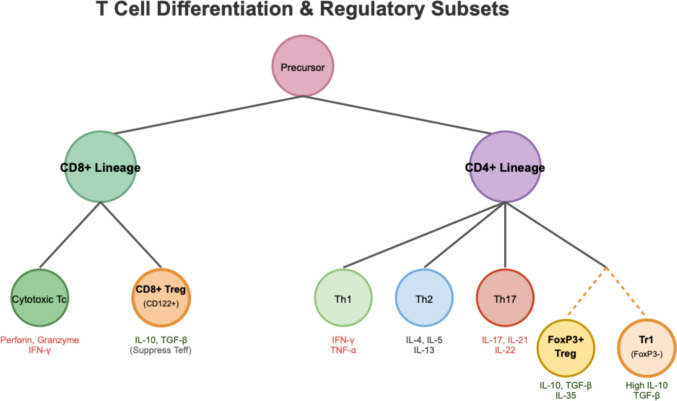
Regulatory T cell subsets and their immunomodulatory mechanisms in acute neuroinflammation. This schematic illustrates the differentiation of T lymphocyte precursors into distinct effector and regulatory lineages, highlighting both classical FOXP3⁺ and non-FOXP3 regulatory T cell subsets that shape immune responses in acute neuroinflammation. CD8⁺ lineage: Naïve CD8⁺ T cells (left branch) differentiate into cytotoxic effector T cells that produce perforin, granzymes, and interferon-γ (IFN-γ) or into CD8⁺ regulatory T cells (CD8⁺ Tregs), a non-FOXP3 suppressive subset characterized by CD122 expression. CD8⁺ Tregs inhibit effector T cell proliferation and dampen neuroinflammation through IL-10- and TGF-β-dependent mechanisms. CD4⁺ lineage: CD4⁺ T cell precursors (right branch) differentiate into proinflammatory effector subsets (Th1 and Th17) or regulatory populations. The regulatory branch diverges into classical FOXP3⁺ regulatory T cells (Tregs), which secrete IL-10, TGF-β, and IL-35 and contribute to blood–brain barrier (BBB) stabilization, and type 1 regulatory T cells (Tr1 cells), a distinct FOXP3-negative regulatory subset that mediates potent immunosuppression predominantly through high-level IL-10 secretion. Functional impact: The balance between FOXP3⁺ Tregs and non-FOXP3 regulatory populations (Tr1 cells and CD8⁺ Tregs) versus proinflammatory effector cells (Th1, Th17, and cytotoxic T cells) determines the neuroimmune milieu and critically influences whether acute neuroinflammation culminates in secondary tissue injury or functional recovery

In addition to canonical FOXP3⁺ regulatory T cells, other regulatory T cell populations may contribute to immune modulation in neuroinflammatory settings. Type 1 regulatory T (Tr1) cells are FOXP3-negative, IL-10-producing suppressive T cells, while CD8⁺ regulatory T cells represent a distinct population capable of limiting effector T cell activation and cytokine production. Although these subsets are less well characterized in acute brain injury, they provide important context for the broader regulatory landscape illustrated in Fig. 2 [67, 69, 70].

Table 1 provides a detailed overview of these T cell subsets, associated cytokines, and their respective roles in neuroinflammatory pathophysiology.

**Table 1:** T cell subsets and associated mediators in acute neuroinflammation

T cell subset/pathway	Key mediators	Primary function	Disease context	Effect on neuroinflammation	References
Th1	IFN-γ, TNF-α	Proinflammatory effector response	Stroke, TBI, ICH, SAH	Microglial activation, BBB disruption, neuronal injury	Sun et al. 2018 [71]; Sonar et al. 2017 [12]; Rahman et al. 2018 [10]; Jayaraman et al. 2005 [72]
Th17	IL-17	Proinflammatory response with neutrophil recruitment	Stroke, MS	Increased BBB permeability, neutrophil infiltration	Rahman et al. 2018 [10]; Shi et al. 2022 [11]
Treg (FOXP3⁺)	IL-10, TGF-β, AREG	Anti-inflammatory and tissue repair	Stroke, TBI, ICH	Suppresses effector T cells, stabilizes BBB, promotes repair	Zhou et al. 2023 [20]; Norden et al. 2014 [73]; Deng et al. 2023 [23]; Liu et al. 2023 [16]; Ito et al. 2019 [27]
Th2 (contextual)	IL-4, IL-5, IL-13	Anti-inflammatory and reparative	Limited data	May promote M2 microglial activation	Cherry et al. 2014 [74]; Gadani et al. 2015 [75]
T cell trafficking	ICAM-1, VCAM-1	Adhesion and transmigration	MS, stroke, TBI	Facilitates T cell entry into CNS	Steffen et al. 1994 [76]; Engelhardt and Ransohoff 2005 [77]; Santos-Lima et al. 2022 [78]
Chemokine signaling	CXCL12/CXCR4, CCL17	Cell migration and recruitment	Stroke, ICH	Directs T cell and Treg migration	Huang et al. 2013 [79]; Deng et al. 2023 [23]
Matrix remodeling	MMPs	Extracellular matrix degradation	Stroke, TBI	Facilitates BBB breakdown and infiltration	Hadass et al. 2013 [80]
Treg expansion	IL-2/IL-2Rα	Treg proliferation	Stroke	Enhances immunoregulation	Zhang et al. 2018 [81]

Table 1 summarizes the key T cell subsets and associated mediators involved in acute neuroinflammation, highlighting their functional roles and contributions to CNS injury and repair.

### Treg Plasticity and Functional Instability in Acute Neuroinflammation

Although Tregs are classically defined by stable FOXP3 expression and immunosuppressive function, accumulating evidence suggests that Tregs exhibit considerable phenotypic plasticity in inflammatory environments [67, 82, 83]. Proinflammatory cytokines including IL-6, IL-1β, TNF-α, and IL-23 can impair Treg stability and promote acquisition of Th1- or Th17-like features through induction of transcription factors such as T-bet and RORγt [83–85]. In certain contexts, destabilized Tregs may produce proinflammatory mediators including IL-17A and IFN-γ, although the significance of these transitions in acute CNS injury remains incompletely understood [83, 86].

This concept may be particularly relevant in acute brain injury, where ischemia, DAMP release, oxidative stress, and BBB disruption generate highly inflammatory microenvironments. Experimental studies in AIS and TBI suggest that these conditions may influence Treg suppressive capacity and alter the balance between protective immune regulation and persistent neuroinflammation [18, 27, 87]. At the same time, emerging evidence indicates that CNS-associated Tregs can acquire tissue-adapted reparative phenotypes involved in oligodendrogenesis, microglial modulation, and tissue remodeling through mediators such as amphiregulin, IL-10, TGF-β, and CCN3 [61, 75, 76].

Recognition of Treg heterogeneity and functional plasticity has important therapeutic implications. Strategies such as low-dose IL-2 or adoptive Treg transfer may ultimately require not only expansion of Treg populations but also stabilization of their regulatory phenotype within the inflammatory CNS microenvironment [67, 88].

### Immunoregulatory Functions of Tregs in CNS Pathology

Tregs, a specialized subset of CD4⁺ T lymphocytes expressing the transcription factor FOXP3, are essential for maintaining immune homeostasis and preventing excessive or misdirected immune responses [82, 89, 90]. Their immunoregulatory functions involve multiple mechanisms, including the secretion of anti-inflammatory cytokines such as IL-10, TGF-β, and amphiregulin (tissue repair factor), the suppression of effector T cell proliferation, and the modulation of antigen-presenting cell (APC) activation [13, 68, 91].

Beyond cytokine-mediated effects, Tregs employ additional contact-dependent and coinhibitory mechanisms that enhance their suppressive potency. These include CTLA-4-mediated downregulation of costimulatory signaling on antigen-presenting cells, neuropilin-1 (NRP1)-associated stabilization of Treg function within inflamed tissues, and direct cell–cell interactions that promote tolerogenic APC phenotypes [91, 92]. Through these mechanisms, Tregs may also induce “infectious tolerance,” whereby local immune regulation propagates broader suppressive responses within the inflammatory milieu [93]. CD25 (IL-2Rα), constitutively expressed at high levels on Tregs, enables preferential IL-2 signaling and is critical for their survival, expansion, and functional competence. Together, these pathways complement cytokine-mediated suppression and reinforce Treg-mediated immune regulation in neuroinflammatory environments [67, 94].

In the CNS, Tregs have emerged as key regulators of both chronic autoimmunity and acute neuroinflammation [15]. In MS, autoimmune encephalitis, and neuromyelitis optica spectrum disorder, impairments in Treg number or suppressive function have been associated with increased disease severity and relapse frequency [95, 96]. Experimental models such as experimental autoimmune encephalomyelitis (EAE) further demonstrate that Treg depletion exacerbates CNS inflammation and demyelination, whereas Treg expansion mitigates disease severity and promotes repair [97]. Although these findings are derived primarily from chronic autoimmune settings, they provide important mechanistic insight into the immunoregulatory capacity of Tregs within the CNS and support their emerging role in modulating acute neuroinflammatory responses following brain injury.

Beyond autoimmunity, Tregs also exert critical protective roles in acute brain injuries [21]. In murine models of AIS, ICH, and TBI, Treg depletion results in larger infarct volumes, more severe BBB disruption, and greater proinflammatory cytokine expression [13, 21]. Conversely, Treg expansion via adoptive transfer, low-dose IL-2 therapy, or anti-CD3 monoclonal antibodies has been associated with improved neurological recovery, reduced leukocyte infiltration, and enhanced BBB stability [13, 22, 81, 98].

### Tissue-Resident and Effector Tregs in the CNS

Although Tregs are primarily generated in the thymus or induced peripherally, they are capable of migrating to inflamed tissues including the CNS where they acquire distinct phenotypic and functional traits [27, 99, 100]. Tissue-resident Tregs in the brain exhibit upregulated FOXP3, elevated expression of activation markers such as CD69, CTLA-4, and IL-10, and a tissue-adapted transcriptional profile that enhances their local suppressive function [27, 101, 102].

A summary of key T lymphocyte markers relevant to CNS inflammation and regulatory function is provided in Table 2.

**Table 2:** T lymphocyte markers and their significance in neuroinflammation

Marker	Cell type	Significance in neuroinflammation	Disease context	References
CD8	Cytotoxic T cells	Associated with long-term neurological impairment	TBI, ICH	Shan et al. 2024 [103]
CD25	Activated T cells, Tregs	High expression on Tregs; critical for IL-2 responsiveness	Stroke, TBI, ICH	Sakaguchi et al. 1995 [7]; Boyman and Sprent 2012 [94]
CD69	Activated T cells	Early activation marker indicating local T cell activation	AIS, TBI	Yu et al. 2018 [102]
CCR6	Th17 cells	Mediates recruitment of Th17 cells to inflamed CNS	MS, stroke	Sonar and Lal 2017 [9]
LFA-1	T cells	Binds to ICAM-1, enabling T cell extravasation into CNS	Stroke	Engelhardt and Ransohoff 2005 [77]
γδ TCR	γδ T cells	Subset of T cells involved in early immune response after injury	TBI	Shichita et al. 2009 [104]; Sun et al. 2018 [71]
IL-10R	Multiple cell types	Mediates anti-inflammatory signaling downstream of IL-10 and contributes to Treg-mediated immune regulation	All neuroinflammatory conditions	Zhou et al. 2023 [20]

These CNS-resident Tregs often display an oligoclonal TCR repertoire, suggesting selective expansion in response to CNS-specific antigens [27, 99, 105]. Experimental models of stroke and multiple sclerosis demonstrate that Tregs undergo clonal proliferation upon entry into the injured CNS, particularly in perivascular and leptomeningeal spaces [22, 27]. This expansion corresponds with reduced infiltration of effector T cells, microglial deactivation, and improved neurological outcomes [13, 42].

Beyond adaptive specificity, CNS-resident Tregs functionally modulate local immune and glial cells. Tregs promote the polarization of microglia and infiltrating macrophages toward an M2-like phenotype, characterized by decreased production of IL-1β and TNF-α and increased expression of arginase-1 and insulin-like growth factor-1 (IGF-1), factors associated with tissue repair and debris clearance [42, 106, 107].

Tregs also modulate astrocyte reactivity, limiting pathological GFAP overexpression and shifting astrocytes away from proinflammatory phenotypes. These changes are mediated via TGF-β signaling and appear to reduce astrocyte-derived cytokine and chemokine release [108]. Furthermore, Tregs enhance the expression of tight junction proteins including occludin, claudin-5, and ZO-1 in BMECs, thereby reinforcing BBB integrity and mitigating immune cell infiltration into the parenchyma [13, 27, 109].

Together, these studies highlight the multifaceted roles of tissue-adapted Tregs in shaping the neuroimmune milieu following CNS injury. Their capacity to interact with endothelial cells, astrocytes, and microglia highlights their therapeutic potential as immune orchestrators in acute brain injury [27].

### Treg-Centered Cellular Crosstalk in Neuroinflammation

The outcome of neuroinflammation depends on the finely tuned interactions between innate immune cells, particularly neutrophils and microglia, and the adaptive T cell response, including Tregs. These interactions define the balance between tissue damage and immune resolution following CNS injury [110, 111].

Neutrophils are among the earliest immune cells to infiltrate the CNS after stroke, TBI, or ICH, arriving within hours of BBB disruption [112–114]. While traditionally viewed as acute-phase responders, they actively participate in T cell polarization by secreting cytokines such as TNF-α, IL-1β, IL-12, and TGF-β [115, 116]. These signals promote differentiation of naïve CD4⁺ T cells into Th1, Th17, or Treg subsets depending on the local cytokine context [116, 117]. For example, TGF-β-producing neutrophils can facilitate Treg induction, whereas IL-12-dominant environments favor Th1 skewing [118, 119].

Neutrophils can also act as APCs, expressing MHC class II, CD80, and CD86, directly influencing T cell activation and effector function [120–122]. This role, reported in multiple sclerosis and autoimmune encephalitis, has also been observed in AIS and ICH, where neutrophil-driven antigen presentation contributes to delayed adaptive immune activation [33, 120, 123, 124].

Beyond classical APC roles, neutrophils release neutrophil extracellular trap (NET) web-like chromatin structures embedded with histones and proteases [125–127]. NETs amplify T cell responses by lowering activation thresholds and fostering Th1/Th17 polarization [125, 126, 128]. However, excessive NET formation exacerbates BBB breakdown, endothelial injury, and microglial activation, contributing to sustained inflammation and secondary tissue damage [125, 126, 129]. Notably, IL-1β and IFN-γ promote NET formation [130], and Tregs suppress these cytokines, suggesting a potential indirect role for Tregs in modulating NET release during neuroinflammation [131].

Microglia, the resident immune sentinels of the CNS, sustain longer-term interactions with T cells [132, 133]. Activated microglia upregulate MHC-II, CD80, CD86, and CD40, enabling them to present CNS antigens and modulate T cell differentiation [134]. Microglial-derived cytokines, such as IL-12 and IL-23, promote Th1 and Th17 polarization, leading to BBB permeability and chronic inflammation [128, 133, 135]. Conversely, anti-inflammatory microglia (M2-like phenotype) secrete TGF-β and IL-10, supporting Treg expansion and stability [136, 137].

Importantly, Tregs reciprocally modulate microglial function, shifting them from a proinflammatory M1 state to a reparative M2-like phenotype [24, 136, 137]. Through the secretion of TGF-β, IL-10, and amphiregulin (AREG), Tregs suppress microglial production of TNF-α, IL-1β, and reactive oxygen species, thereby reducing neuronal apoptosis and promoting tissue recovery [24, 66, 136]. Treg depletion models show prolonged microglial activation, increased lesion volumes, and worsened behavioral outcomes, whereas Treg augmentation attenuates microgliosis and promotes neuroprotection [21].

**Fig. 3:**
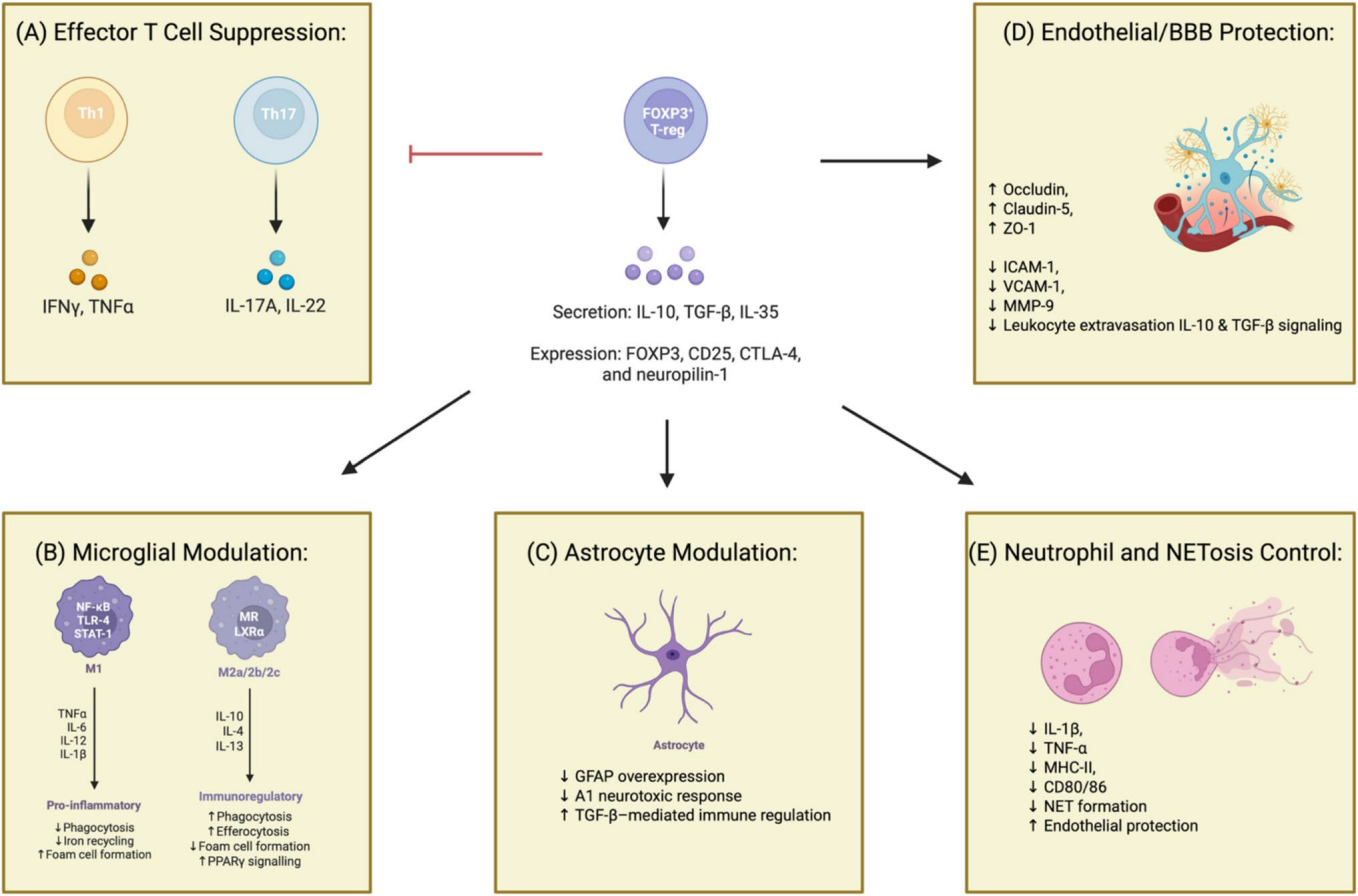
Pleiotropic neuroprotective mechanisms of regulatory T cells within the neurovascular unit. This schematic illustrates the multimodal immunomodulatory functions of FOXP3⁺ regulatory T cells (Tregs) in the setting of acute brain injury. Tregs secrete anti-inflammatory cytokines (IL-10, TGF-β, and IL-35) and express key surface molecules (CTLA-4, neuropilin-1, and CD25) that collectively promote the transition from inflammatory injury to immune resolution and tissue repair. **A** Effector T cell suppression: Tregs inhibit the proliferation and cytokine production of proinflammatory Th1 and Th17 cells, suppressing the release of IFN-γ, TNF-α, IL-17A, and IL-22, thereby limiting secondary autoimmune-like neurotoxicity. **B** Microglial modulation: Tregs drive a phenotypic shift in microglia from a proinflammatory M1 state, characterized by NF-κB and TLR-4 signaling, toward a reparative M2 phenotype. This transition enhances phagocytosis and efferocytosis, promotes debris clearance, and attenuates oxidative and cytokine-mediated injury. **C** Astrocyte modulation: Treg-derived TGF-β suppresses reactive astrogliosis by limiting A1 neurotoxic astrocyte polarization and reducing GFAP overexpression, while supporting neurotrophic and immunoregulatory astrocyte functions. **D** Endothelial and blood–brain barrier (BBB) protection: Tregs preserve BBB integrity by upregulating tight junction proteins (occludin, claudin-5, and ZO-1) and downregulating endothelial activation markers (ICAM-1, VCAM-1) and matrix metalloproteinase-9 (MMP-9), thereby restricting leukocyte adhesion and extravasation. **E** Neutrophil and NETosis control: Tregs suppress neutrophil recruitment and neutrophil extracellular trap (NET) formation, reducing vascular inflammation, endothelial injury, and collateral tissue damage within the neurovascular unit. Abbreviations: BBB, blood–brain barrier; GFAP, glial fibrillary acidic protein; MMP-9, matrix metalloproteinase-9; NET, neutrophil extracellular trap; NVU, neurovascular unit; ZO-1, zonula occludens-1. Image created using BioRender.

This reciprocal cellular crosstalk where neutrophils and microglia shape T cell fate and Tregs reprogram innate immune responses defines the immunological trajectory of CNS injury [33, 114]. A deeper understanding of these interactions is essential for identifying therapeutic checkpoints to enhance Treg-mediated resolution while mitigating pathological inflammation (Fig. 3).

The interactions described above unfold in a highly ordered temporal sequence. In the first 0–24 h, neutrophils dominate the CNS immune landscape, releasing TNF-α, IL-1β, IL-12, ROS, and forming NETs that lower T cell activation thresholds and promote Th1/Th17 polarization. Between 24 and 72 h, Tregs first enter the CNS as BBB permeability increases; their IL-10 and TGF-β secretion suppresses NETosis, reduces neutrophil chemotaxis, and begins shifting microglia away from a proinflammatory M1 state. During days 3–7, Tregs reach peak activity and drive M1 → M2 phenotypic conversion in microglia and infiltrating macrophages through IL-10/GSK3β/PTEN and TGF-β/Smad2/3 pathways, marking the onset of the resolution phase. After 7 days, tissue-resident Tregs sustain IL-10 production, support angiogenesis and synaptic remodeling, and maintain long-term neuroprotection.

**Fig. 4:**
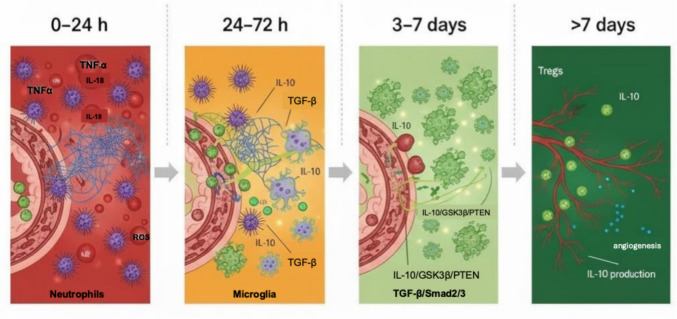
Temporal dynamics of regulatory T cell (Treg)–innate immune interactions following acute brain injury. This schematic depicts the sequential and time-dependent interactions between regulatory T cells (Tregs) and innate immune populations across the hyperacute, subacute, and recovery phases following acute brain injury. 0–24 h (hyperacute phase): Neutrophils predominate within the injured central nervous system (CNS), accompanied by extensive neutrophil extracellular trap (NET) formation and the release of proinflammatory mediators, including tumor necrosis factor-α (TNF-α), interleukin-1β (IL-1β), and reactive oxygen species (ROS). These events coincide with early blood–brain barrier (BBB) disruption and limited Treg presence within the CNS. 24–72 h (early subacute phase): Tregs begin to infiltrate the CNS, where their secretion of IL-10 and transforming growth factor-β (TGF-β) suppresses excessive NETosis, attenuates neutrophil chemotaxis, and initiates the downregulation of proinflammatory, M1-polarized microglia. 3–7 days (resolution phase): Treg activity peaks, driving the phenotypic transition of microglia and macrophages from an M1 to an M2 reparative state through IL-10/GSK3β/PTEN and TGF-β/Smad2/3 signaling pathways, thereby promoting inflammation resolution and tissue repair. > 7 days (recovery and remodeling phase): Tissue-resident Tregs support long-term repair by sustaining IL-10 production, stabilizing the neurovascular environment, and promoting angiogenesis, synaptic remodeling, and durable neuroprotection. Abbreviations: BBB, blood–brain barrier; CNS, central nervous system; GSK3β, glycogen synthase kinase 3 beta; IL, interleukin; NET, neutrophil extracellular trap; PTEN, phosphatase and tensin homolog; ROS, reactive oxygen species; TGF-β, transforming growth factor beta; Treg, regulatory T cell.

Together, this timeline demonstrates that neutrophils and microglia shape the early inflammatory environment, while Tregs progressively reprogram innate immune responses to guide the transition from acute inflammation to immune resolution and repair (Fig. 4).

## Pathophysiological Roles of Tregs Across the Spectrum of Acute Brain Injury

### Acute Ischemic Stroke

AIS is a leading cause of long-term disability and mortality worldwide [138]. While thrombolytic therapy and mechanical thrombectomy have significantly improved stroke management, many patients remain ineligible for these interventions, particularly those with medium and distal vessel as well as posterior circulation strokes or delayed hospital arrival [139]. Given these limitations, there is growing interest in targeting immune-mediated secondary injury as a therapeutic strategy in AIS [140].

#### Th1 and Th17 Cells in AIS Pathogenesis

Th1 and Th17 cells are key drivers of “post-stroke” neuroinflammation, contributing to worsened neurological outcomes by exacerbating secondary injury mechanisms in the ischemic brain [13, 33, 132]. Th1 cells promote proinflammatory responses through the release of IFN-γ, which amplifies microglial activation, disrupts the BBB, and contributes to neuronal apoptosis [10, 13, 71]. Increased Th1 activity following AIS has been associated with heightened leukocyte infiltration, prolonged neuroinflammation, and increased infarct volume, further worsening functional recovery [13, 141].

Th17 cells, characterized by their production of interleukin-17A (IL-17A), have been implicated in post-stroke neuroinflammation and vascular pathology [104, 132]. IL-17A is associated with endothelial dysfunction and vascular inflammation, contributing to cerebrovascular instability and increased stroke risk [10, 129]. Following ischemic injury, IL-17A promotes leukocyte recruitment, enhances blood–brain barrier permeability, and sustains a proinflammatory milieu, thereby exacerbating tissue damage and delaying repair [13, 132]. Clinical studies in patients with acute ischemic stroke have demonstrated that elevated IL-17 levels correlate with increased stroke severity and worse neurological outcomes, with emerging evidence suggesting an association with recurrence risk [125, 126, 142].

Importantly, these effects are counterbalanced by regulatory T cells, which suppress Th17 differentiation and IL-17 signaling through IL-10 and TGF-β-mediated pathways. Disruption of this Treg–Th17 equilibrium may therefore represent a critical determinant of post-stroke inflammation. Accordingly, therapeutic strategies aimed at restoring this balance, including modulation of Th17 polarization or enhancement of Treg function, are being actively explored [135, 143, 144].

#### Treg Cells as Modulators of Post-Stroke Inflammation

In contrast to the proinflammatory effects of Th1 and Th17 cells, Tregs play a critical role in counteracting inflammation and promoting “post-stroke” repair. By exerting immunosuppressive and neuroprotective effects, Tregs help regulate the balance between destructive and reparative immune responses following AIS [13, 21, 31, 32].

Tregs mediate their protective function primarily through the secretion of IL-10 and TGF-β, which suppress proinflammatory cytokine production, mitigate excessive microglial overactivation, and preserve BBB integrity [16, 73, 145]. The ability of Tregs to limit excessive microglial activation prevents the unchecked release of neurotoxic mediators such as TNF-α and reactive oxygen species (ROS), thereby reducing neuronal apoptosis and secondary brain injury [13, 42, 61]. In experimental stroke models, Treg depletion has been shown to worsen neurological deficits, increase infarct size, and prolong neuroinflammation, highlighting their crucial role in the resolution of ischemic injury [13, 128].

Beyond direct neuroprotection, Tregs contribute to infarct resolution by modulating the infiltration and polarization of peripheral immune cells. They promote anti-inflammatory macrophage phenotypes (M2-like) while dampening the recruitment of inflammatory monocytes and neutrophils, thereby creating a more favorable environment for angiogenesis, tissue repair, and functional recovery [23, 59, 64].

#### Targeting Th1/Th17/Treg Balance for Stroke Therapy

Emerging research highlights the therapeutic potential of modulating the Th1/Th17/Treg axis to reduce “post-stroke” neuroinflammation and promote functional recovery [146, 147]. Strategies aimed at inhibiting proinflammatory Th1/Th17 responses while enhancing Treg-mediated immune resolution have shown promise in experimental models of AIS [21, 81, 132].

One promising approach involves blocking the CXCL12/CXCR4 signaling pathway, which plays a crucial role in the recruitment of pathogenic T cells into the ischemic brain in the acute phase [148, 149]. CXCL12/CXCR4 inhibition has been shown to reduce T cell infiltration, thereby limiting neuroinflammation and BBB breakdown. This approach has been explored in preclinical stroke models, demonstrating significant reductions in infarct volume and improved neurological outcomes [149].

Mesenchymal stem cell (MSC) therapy has also emerged as a potential intervention for restoring Th17/Treg balance [127]. Studies suggest that umbilical MSC-derived mitochondrial transfer can correct T cell dysfunction, shifting the immune response away from proinflammatory Th17 activity while enhancing Treg-mediated neuroprotection [127, 130]. This process has been linked to improved post-stroke motor function, reduced BBB permeability, and enhanced angiogenesis in experimental models [127, 131].

Another promising strategy involves Treg expansion via low-dose IL-2 therapy, which selectively enhances regulatory T cell populations while minimizing activation of proinflammatory effector T cells [81]. Preclinical data suggest that IL-2 therapy enhances Treg-mediated suppression of microglial overactivation, reduces post-stroke neuroinflammation, and helps maintain BBB integrity [81]. Early-phase clinical trials in neurological disorders have begun to explore the feasibility of low-dose IL-2-based immune modulation, demonstrating expansion of regulatory T cells and supporting its potential translation into targeted immunotherapies for stroke [133, 134].

### Intracerebral Hemorrhage

#### Th1 and Th17 Cells in ICH Pathogenesis

ICH initiates a profound inflammatory response within the brain, contributing to secondary injury, edema formation, and poor neurological outcomes [136]. Among the key immune drivers are Th1 and Th17 cells, which act synergistically to amplify inflammatory cascades. Th1-derived IFN-γ promotes microglial and endothelial activation, facilitating blood–brain barrier disruption and leukocyte infiltration into perihematomal regions [137]. Concurrently, Th17 cells, primarily through IL-17A signaling, enhance vascular permeability, recruit neutrophils, and upregulate matrix metalloproteinases, further destabilizing the neurovascular unit [150, 151]. Together, these pathways exacerbate perihematomal injury, promote neuronal apoptosis, and impair tissue repair [25]. Consistent with these mechanisms, elevated Th1- and Th17-associated cytokines in both experimental models and patients with ICH have been linked to larger hematoma volumes, increased perihematomal edema, and worse neurological outcomes.

#### Treg-Mediated Neuroprotection in ICH

In contrast to proinflammatory T cell subsets, regulatory T cells act as key modulators of post-ICH neuroinflammation. Following hemorrhagic injury, Tregs accumulate in perihematomal regions and contribute to immune resolution. In contrast to proinflammatory T cell subsets, regulatory T cells act as key modulators of post-ICH neuroinflammation [151]. Following hemorrhagic injury, Tregs accumulate in perihematomal regions and contribute to immune resolution [24]. A central mechanism involves the regulation of microglial and macrophage polarization, whereby Treg-derived cytokines promote a shift toward a reparative M2-like phenotype [23]. This transition suppresses proinflammatory mediators such as TNF-α and IL-6, reduces oxidative stress, and supports a neuroprotective environment conducive to recovery [24].

Tregs also exert direct inhibitory effects on microglial activation [24]. By suppressing the production of proinflammatory mediators, they help preserve neuronal viability and limit edema expansion. This immunoregulatory influence further contributes to blood–brain barrier stabilization by reducing endothelial activation and inflammatory signaling, thereby maintaining barrier integrity and limiting secondary infiltration of peripheral immune cells [151].

#### Treg Recruitment via Chemokine Signaling

The beneficial effects of regulatory T cells are further enhanced by chemokine-mediated recruitment to sites of injury. Among these, the CCL17–CCR4 axis plays a critical role in directing Treg trafficking to the perihematomal region following intracerebral hemorrhage [23, 152]. Increased local expression of CCL17 promotes Treg accumulation within the injured brain, where they suppress excessive immune activation, limit leukocyte infiltration, and facilitate neurovascular repair [23, 152]. Experimental studies demonstrate that augmentation of this pathway enhances functional recovery and attenuates neuroinflammation, highlighting chemokine-guided Treg recruitment as a promising therapeutic strategy in ICH [23].

#### Therapeutic Strategies Targeting Tregs in ICH

Several preclinical strategies have been developed to enhance regulatory Treg activity following ICH, yielding promising neuroprotective outcomes. Adoptive transfer of Tregs has been shown to reduce perihematomal inflammation, suppress TLR4/NF-κB signaling, and improve both short- and long-term neurological outcomes in murine models. Mechanistically, transferred Tregs also protect the BBB by downregulating neutrophil-derived MMP-9 and inflammatory cytokines [24].

Cytokine-based expansion strategies represent a promising approach to enhance regulatory T cell activity following intracerebral hemorrhage. Administration of IL-2/anti–IL-2 complexes selectively expands CD25^high^ FOXP3^+^ Tregs while minimizing activation of effector T cells and has been shown to promote white matter repair and functional recovery in experimental models [153]. Although direct evaluation in ICH remains limited, this strategy is being explored for brain-selective immunomodulation.

Chemokine-guided recruitment provides a complementary strategy. Augmentation of the CCL17–CCR4 axis enhances Treg infiltration into the injured brain, promotes microglial and macrophage polarization toward reparative phenotypes, and reduces edema and neuroinflammation in ICH models [23, 154].

Pharmacologic modulation of key immunoregulatory pathways can further amplify Treg responses. Inhibition of mTOR signaling with rapamycin increases FOXP3^+^ Treg populations and shifts the cytokine milieu toward an anti-inflammatory profile, while PD-L1-mediated signaling enhances regulatory immune responses and suppresses Th1/Th17-driven inflammation, contributing to improved neurobehavioral outcomes and preservation of blood–brain barrier function [155, 156].

Finally, amplification of IL-10 signaling, a central effector pathway of Tregs, further supports blood–brain barrier stability, attenuates microglial activation, and promotes neuroprotection. These effects may be mediated through endogenous Treg activity or IL-10-based cellular therapies [157].

Collectively, these strategies target complementary aspects of Treg biology, including expansion, trafficking, functional polarization, and effector signaling, and may be leveraged in combination to enhance therapeutic efficacy in future translational studies.

### Cerebral Aneurysms and Aneurysmal Subarachnoid Hemorrhage (aSAH)

Following aneurysmal subarachnoid hemorrhage, patients experience an initial hemorrhagic insult followed by secondary brain injury driven by neuroinflammation [158, 159]. This sustained inflammatory response contributes to delayed cerebral ischemia, a major determinant of poor neurological outcomes [160, 161]. Early immune activation is characterized by microglial polarization, driven by pattern recognition receptors such as toll-like receptors and nucleotide-binding oligomerization domain–like receptors, which detect vascular injury and initiate inflammatory signaling cascades [162, 163]. Activation of these pathways promotes the release of proinflammatory mediators, including IL-1β, MCP-1, matrix metalloproteinases, and TNF-α from microglia, astrocytes, endothelial cells, and infiltrating immune cells, amplifying neuroinflammation and tissue injury [164]. In contrast, alternatively activated microglia exert neuroprotective effects through the production of IL-10, supporting tissue repair, angiogenesis, and blood–brain barrier stabilization [20, 158].

#### T Lymphocytes in Aneurysm Formation and SAH Progression

T lymphocytes are central to the initiation and progression of inflammation in cerebral aneurysm formation and aSAH pathology [158, 165]. They contribute to aneurysm development by secreting IL-1β, IL-6, TNF-α, and endothelin-1, which induce chronic vascular inflammation and extracellular matrix degradation [165–167]. TNF-α plays a pivotal role in intracranial aneurysm pathogenesis by activating NF-κB signaling, which upregulates proinflammatory cytokines such as IL-1α, IL-1β, and IL-6 [167]. These signals not only induce apoptosis of vascular smooth muscle cells (VSMCs) leading to loss of vessel wall integrity but also contribute to a pathological shift in T cell polarization.

Specifically, proinflammatory cytokines such as IL-6 and TNF-α shape the adaptive immune response by promoting differentiation of naïve CD4^+^ T cells toward the Th17 lineage while simultaneously impairing regulatory T cell stability and function [143]. This shift is mediated, in part, through disruption of FOXP3 expression and signaling pathways that maintain Treg identity, thereby favoring a proinflammatory phenotype [143]. Within the context of cerebral aneurysm disease, this cytokine-driven imbalance contributes to vascular smooth muscle cell apoptosis, extracellular matrix degradation, and progressive vessel wall weakening [165, 167]. Collectively, this Treg–Th17 disequilibrium represents a critical axis of maladaptive immunity in aneurysm pathophysiology.

Sawyer et al. [165] demonstrated that T lymphocytes regulate extracellular matrix remodeling through cytokine secretion, thereby altering VSMC phenotype. This contractile-to-synthetic transition results in an increased production of inflammatory mediators at the expense of vascular integrity. Ultimately, this structural weakening of the aneurysmal vessel wall renders it more prone to abnormal dilation and rupture, further increasing the risk of aSAH [165].

Lymphocyte infiltration also plays a key role in the pathogenesis of cerebral vasospasm following SAH, with CD4^+^ T lymphocytes accumulating within cerebral arteries in the early phase post-SAH [168]. In both human and experimental models, T cell infiltration peaks within the first 3 days post-SAH, coinciding with an increase in peripheral T cell activation and heightened inflammatory responses [169, 170]. Elevated Th1 and Th17 responses correlate with increased vasospasm risk and worse clinical outcomes, while Th2 levels decline, reflecting a shift toward a proinflammatory state [25, 171].

#### Tregs in aSAH: Modulating the Post-Hemorrhagic Immune Response

Tregs play a crucial immunosuppressive role in aSAH by modulating post-hemorrhagic inflammation and facilitating neurological recovery. However, in aSAH patients, the proliferative capacity of Tregs is significantly impaired, correlating with increased T lymphocyte adhesion in the peripheral circulation. FOXP3^+^ Tregs function by secreting IL-10, TGF-β, and IL-35, which enhance immune tolerance, reduce leukocyte infiltration, and mitigate secondary brain injury [20].

In vivo studies reveal that Treg cell depletion worsens SAH outcomes, increasing susceptibility to angiotensin II-induced aneurysm rupture. Conversely, Treg reconstitution restores immunoinflammatory balance, suppressing aneurysm progression and reducing vascular inflammation [171]. Tregs infiltrate the brain within 1 day post-SAH, where they suppress neuroinflammation via IL-10 secretion, reducing neuronal apoptosis, BBB breakdown, and excessive microglial activation [20]. These findings highlight the therapeutic potential of Treg-based interventions for preventing aSAH-induced secondary brain injury and vasospasm-related complications [25].

CD4^+^ T cells play a dual role in the aftermath of aSAH, driving inflammatory injury while also providing a regulatory function through Tregs [25]. The impairment of Treg-mediated immune suppression following aSAH correlates with increased inflammation, vascular instability, and poor clinical outcomes. These insights suggest that therapeutic strategies aimed at enhancing Treg responses such as IL-2-based expansion therapy or adoptive Treg transfer may offer new approaches to mitigating aSAH progression and preventing secondary complications. Together, these findings highlight how an imbalance between proinflammatory and regulatory T cell responses contributes to poor outcomes in aSAH, reinforcing the therapeutic potential of Treg-targeted interventions in future clinical studies [25, 172].

### TBI and T Cell-Mediated Inflammation

TBI is a leading cause of long-term neurological disability and mortality worldwide, affecting individuals across all age groups [173]. Patients with moderate-to-severe TBI (msTBI) often require intensive care unit (ICU) admission for neuromonitoring, surgical intervention, and management of secondary complications [174]. The severity of TBI is closely linked to long-term outcomes, and accumulating evidence associates TBI with an increased risk of post-traumatic dementia. Moreover, repetitive TBIs have been implicated in the development of chronic traumatic encephalopathy, a progressive neurodegenerative condition characterized by sustained neuroinflammation, tauopathy, and cognitive decline [175].

#### T Cells in the Inflammatory Cascade of TBI

The immune response to TBI plays a dual role, contributing to both repair and neurodegeneration [176]. Following brain trauma, disruption of the BBB facilitates the infiltration of peripheral immune cells, including CD4⁺ and CD8⁺ T lymphocytes [177]. These cells further amplify the post-injury inflammatory cascade through cytokine production and cell-to-cell interactions [176, 178]. Activated T cells release proinflammatory mediators such as IL-17 and IFN-γ, which can contribute to debris clearance and the initiation of tissue repair [178, 179]. However, when these pathways remain persistently activated, they contribute to maladaptive inflammation, promoting microglial overactivation, astrogliosis, and neuronal apoptosis [176, 178].

T cells also engage in reciprocal crosstalk with CNS-resident cells, including microglia and astrocytes. In response to T cell-derived cytokines such as IFN-γ and IL-17, these glial cells adopt a proinflammatory phenotype, thereby sustaining a neurotoxic environment that contributes to secondary neuronal injury and chronic white matter degeneration [180]. This persistent inflammatory state has been implicated in long-term deficits in memory, executive function, and sensorimotor performance commonly observed following TBI. Notably, chronic activation of Th1 and Th17 cells has been linked to ongoing neuroinflammation and the development of post-TBI neurodegeneration, correlating with worse long-term neurological outcomes [181].

#### Tregs in TBI: Modulating Neuroinflammation and Repair

Tregs play a critical role in modulating the neuroinflammatory response following TBI [103]. Both clinical and preclinical studies have documented dynamic changes in Treg numbers and function following TBI. Patients with msTBI exhibit early fluctuations in circulating Tregs, and higher levels of Tregs during the first 14 days post-injury have been correlated with better neurological outcomes [182].

In murine models, TBI induces an early rise in brain-infiltrating Tregs, particularly those expressing ST2, the IL-33 receptor subunit, suggesting involvement of IL-33/ST2 signaling in Treg-mediated protection [183]. Depletion of Tregs after controlled cortical impact (CCI) leads to increased T cell infiltration, upregulated IFN-γ, and exacerbated reactive astrogliosis, reinforcing their anti-inflammatory role [18]. Conversely, adoptive transfer of human Tregs after injury attenuates chronic neuroinflammation, although it does not significantly improve blood–brain barrier integrity in experimental models [184].

Tregs exert their protective effects in part by inhibiting microglial and astrocyte activation, modulating the balance between Th17 and Treg phenotypes, and reducing cytokine-driven tissue damage [145, 185]. Notably, pharmacological expansion of Tregs via IL-2/anti-IL-2 complexes has been shown to increase FOXP3^+^ Treg populations in the brain, reduce brain edema, and improve neurological function [145]. Similarly, astrocyte-targeted IL-2 gene therapy selectively expands brain-resident Tregs and induces an anti-inflammatory shift in microglia without causing systemic immunosuppression [153].

In addition to their immunosuppressive functions, regulatory T cells may also contribute to tissue repair and regeneration. Experimental studies have demonstrated that Tregs secrete factors such as IL-10, TGF-β, and CCN3, which support oligodendrogenesis and white matter recovery in models of central nervous system injury [61, 63]. Although these findings are not specific to traumatic brain injury, they suggest a potential role for Tregs in promoting repair processes following TBI.

Combination approaches, such as coadministration of Tregs with mesenchymal stem cells, have shown enhanced immunomodulatory effects in experimental models, although therapeutic efficacy appears to depend on timing, dosing, and injury context [186] (Table 3).

**Table 3:** Comparative overview of Treg functions across major acute brain injuries

Feature	Acute ischemic stroke (AIS)	Intracerebral hemorrhage (ICH)	Aneurysmal subarachnoid hemorrhage (aSAH)	Traumatic brain injury (TBI)
Primary pathological trigger	Arterial occlusion → ischemia, excitotoxicity	Hematoma mass effect + blood toxicity	Extravasated blood in cisterns; early brain injury	Mechanical disruption, axonal injury
Early immune environment (0–24 h)	Rapid Th1/Th17 influx; microglial activation; high ROS	Neutrophils + inflammatory monocytes dominate	Systemic immune depression + vascular inflammation	Neutrophils early; strong M1 microglial activation
Timing of Treg infiltration	24–72 h; peak ~ day 5	24–48 h; strong recruitment via CCL17	Reduced proliferative capacity in peripheral blood; CNS entry ~ 24–72 h	3–7 days; peak in subacute period
Key Treg mechanisms	IL-10/TGF-β suppress microglial overactivation; BBB repair; reduce infarct progression	IL-10/GSK3β/PTEN-mediated M1 → M2 conversion; hematoma toxicity suppression	IL-10 reduces vasospasm, endothelial injury; modulates early brain injury	IL-10/TGF-β reduce chronic microglial activation; promote synaptic repair
Effect on microglia/macrophages	Shift from proinflammatory M1 → M2-like repair phenotype	Strong M1 → M2 polarization; enhances hematoma clearance	Reduces microglial NF-κB activity; supports vascular stabilization	Limits persistent M1 microglial activation implicated in long-term deficits
Effect on neutrophils/NETs	Suppresses NETosis; reduces secondary BBB injury	Moderately reduces neutrophil-driven oxidative damage	Limits early neutrophil-mediated vascular inflammation	Modest effect; reduces neutrophil ROS and cytokines
Effect on BBB	Strong stabilization via tight junction restoration	Moderate stabilization; attenuates thrombin-induced permeability	Protects endothelial cells during early brain injury	Reduces chronic BBB leakiness
Downstream neuroprotective effects	Smaller infarcts, improved functional recovery	Less perihematomal edema, better neurobehavioral outcomes	Reduced vasospasm, lower DCI risk	Improved cognition, reduced chronic inflammation
Therapeutic Treg strategies studied	IL-2/IL-2 complex; adoptive Treg transfer; CXCL12 modulation	CCL17-driven recruitment; IL-10 pathway targeting	IL-2 expansion; adoptive transfer models; endothelial protection	IL-2 therapy; exosome-based Treg delivery
Shared mechanisms across all injuries	IL-10/TGF-β immunosuppression; M1 → M2 shift; microglial modulation; NET suppression; BBB stabilization			
Key disease-specific distinctions	High Th17 involvement; reperfusion injury context	Hematoma toxicity uniquely requires M2 phagocytic support	Unique vasospasm/DCI biology with impaired Treg expansion	Long-term inflammation central; chronic microglial overdrive

## Therapeutic Strategies Targeting Tregs in Neuroinflammation

Multiple complementary approaches expand, recruit, or potentiate Tregs to resolve neuroinflammation in acute brain injury.

### Low-Dose IL-2-Based Treg Expansion

Among the most well-studied approaches is low-dose IL-2 therapy, which preferentially expands Tregs by exploiting their high expression of the IL-2Rα (CD25) chain. This approach enhances Treg suppressive function while minimizing activation of effector T cell [187]. In AIS, low-dose IL-2 has been shown to preserve BBB integrity, reduce infarct volume, and promote neurogenesis and angiogenesis, primarily through increased IL-10 secretion and suppression of proinflammatory Th17 response [31, 32, 187]. In aSAH, low-dose IL-2 administration post-injury increased peripheral and brain-infiltrating Tregs, reduced neuronal apoptosis, and improved motor outcomes, likely via enhanced IL-10 secretion and inhibition of inflammatory cascades [20, 187]. Together, these findings suggest that Treg expansion via low-dose IL-2 or IL-2-based biologics represents a promising immunotherapeutic strategy to modulate neuroinflammation and improve outcomes in acute brain injuries. Despite its promise, low-dose IL-2 therapy requires careful dose optimization to avoid off-target activation of effector T cells.

### Adoptive Transfer of Tregs in Neuroinflammatory Diseases

Another promising strategy involves adoptive transfer of regulatory T cells, in which Tregs are isolated, expanded ex vivo, and reinfused to enhance immune regulation and tissue repair. In experimental models of acute ischemic stroke, Treg transfer reduces infarct size, suppresses microglial activation, and improves long-term neurological outcomes, in part through preservation of blood–brain barrier integrity and secretion of anti-inflammatory and reparative mediators such as IL-10 and amphiregulin [21, 27, 31].

In traumatic brain injury, adoptive Treg therapy has been associated with reduced lesion volume, attenuation of chronic neuroinflammation, and enhanced synaptic remodeling and neurogenesis. These effects are thought to be mediated, at least in part, through IL-10-dependent modulation of microglial activation and promotion of a reparative immune environment [103, 184].

Overall, adoptive Treg therapy offers a targeted, cell-based strategy to re-establish immune homeostasis and protect against secondary injury across acute CNS disorders [21, 31]. However, this approach is limited by challenges related to cell sourcing, ex vivo expansion, and maintenance of phenotypic stability, which may impact its clinical scalability.

### Targeting T Cell Infiltration and BBB Protection

Excessive T cell infiltration into the CNS is a key driver of neurovascular injury in acute brain disorders such as AIS, ICH, and TBI [188–190]. T cells migrate across the BBB through upregulated endothelial adhesion molecules especially ICAM-1 and VCAM-1 in response to inflammatory cues [190, 191].

In experimental models of AIS and ICH, blocking ICAM-1 or VCAM-1 has been shown to reduce T cell and other leukocyte trafficking into the CNS, limit secondary neuroinflammation, and preserve BBB integrity [112, 188, 192]. More recently, Tregs themselves have been shown to suppress ICAM-1 and VCAM-1 expression on endothelial cells, thereby exerting indirect control over T cell infiltration [193, 194]. For instance, adoptive transfer of Tregs in stroke models was associated with reduced endothelial activation and downregulation of adhesion molecule expression, contributing to improved vascular function [21, 193, 195].

In addition to adhesion molecules, chemokine signaling pathways, particularly the CXCL12/CXCR4 axis, play an important role in guiding T cell migration into the brain parenchyma following injury [79, 149, 152, 196]. In stroke and ICH models, pharmacologic inhibition of CXCR4 (e.g., with AMD3100, plerixafor) has been shown to reduce T cell accumulation in perivascular spaces, dampen inflammatory cascades, and improve long-term neurological outcomes [152, 196]. Overall, modulating adhesion molecule expression and chemokine-driven T cell trafficking, either directly through pharmacologic agents or indirectly via Treg-based interventions, holds significant therapeutic potential for preserving BBB integrity and reducing neuroinflammation [79, 188, 192, 193, 195].

### Matrix Metalloproteinase Inhibition as a Neuroprotective Strategy

MMPs are key enzymes involved in BBB degradation and neuroinflammatory damage following acute brain injury [197]. Elevated MMP activity has been linked to increased T cell-mediated neuroinflammation, particularly through enhanced BBB permeability and leukocyte extravasation [144, 198]. MMP inhibitors have been shown in preclinical models to prevent leukocyte-driven neurovascular damage, reduce secondary injury progression, and preserve neuronal integrity in preclinical models of stroke and TBI [199, 200].

Therapeutic strategies targeting Tregs and their associated immune pathways provide a multifaceted approach to managing acute neuroinflammation. By expanding Treg populations (IL-2 therapy, adoptive Treg transfer), limiting harmful immune cell infiltration (ICAM-1/VCAM-1 blockade, CXCL12-CXCR4 blockade), and protecting the BBB (MMP inhibition), these approaches offer novel opportunities to improve outcomes in CNS disorders [21]. Ongoing clinical trials and translational research are essential to refining these interventions for human application, ensuring optimized dosing, delivery methods, and patient selection criteria for maximum therapeutic efficacy [201].

MMPs are zinc-dependent endopeptidases that play a pivotal role in extracellular matrix remodeling, basement membrane degradation, and BBB disruption following acute brain injury [197]. In the context of stroke and TBI, MMP-2 and MMP-9 are rapidly upregulated in endothelial cells, infiltrating leukocytes, and activated microglia, promoting T cell transmigration and paracellular leakage [198]. Elevated MMP activity facilitates leukocyte extravasation into the CNS, amplifying neuroinflammation, neuronal damage, and cerebral edema. In preclinical models of AIS, pharmacological inhibition of MMP-9 (e.g., using minocycline or specific MMP inhibitors) preserved BBB integrity, reduced immune cell infiltration, and improved neurological outcomes [202].

In TBI, MMP inhibition similarly attenuated glial activation, BBB leakage, and neuronal apoptosis, with some evidence suggesting that MMPs also modulate the polarization and recruitment of peripheral T cells [80]. Emerging studies indicate that Tregs can suppress MMP-9 expression indirectly, via IL-10-mediated inhibition of NF-κB signaling, further supporting the interplay between MMP activity and regulatory immune mechanisms [33, 203, 204].

Some of the key clinical trials targeting Tregs in neurological disease are listed in Table 4.

**Table 4:** Summary of key clinical trials targeting regulatory T cells in neurological diseases

Disease context	Trial name/ID	Therapeutic strategy	Phase	Status and key findings
Aneurysmal SAH	LIL-SAH (NCT03085819)	Low-dose IL-2 (Proleukin)	II	Completed. Demonstrated safety and selective expansion of Tregs in patients with subarachnoid hemorrhage. Post hoc analysis showed significant reduction in IL-6 levels
ALS	Tregs in ALS (NCT04055623)	Autologous Treg adoptive transfer + IL-2	I	Completed. Infusions were safe and well tolerated. Treg numbers and suppressive function increased. Progression of clinical decline (ALSFRS-R) slowed during the treatment phase compared to washout
ALS	MIROCALS (NCT03039673)	Low-dose IL-2	II	Completed. Met primary safety endpoints. Analysis indicated a significant survival benefit in a subset of patients with specific inflammatory profiles, validating the immunomodulatory approach
ALS	REGALS (NCT05695521)	Allogenic cord-blood derived Tregs (CK0803)	Ib	Recruiting/ongoing. Investigating safety and tolerability of multidose allogenic Treg cells designated with neurotropic homing markers
Alzheimer’s disease	LIL-AD (NCT05468073)	Low-dose IL-2 (COYA 301)	II	Completed. Met primary safety endpoints. Significant expansion of Treg populations observed. Exploratory data suggested cognitive stabilization (ADAS-Cog14) in the 4-week dosing cohort
Acute ischemic stroke	L-DOSE (NCT02053755)	Low-dose IL-2	I/II	Completed. Validated the safety of low-dose IL-2 in diverse autoimmune/inflammatory conditions, establishing dose-dependent Treg expansion kinetics relevant for stroke translation

## Current Limitations and Translational Challenges

Despite the therapeutic promise of Tregs in acute neuroinflammation, the transition from preclinical discovery to clinical application faces substantial biological, methodological, and practical hurdles [13, 60].

### Methodological Hurdles and Biological Gaps

A primary limitation in the field is the incomplete understanding of Treg heterogeneity. While CD4⁺FOXP3⁺ Tregs are the best-characterized subset, emerging data highlight the importance of other regulatory populations, such as CD8⁺CD122⁺ T cells and IL-10-producing Tr1 cells. However, the specific trafficking behaviors, cytokine dependencies, and functional relevance of these noncanonical subsets within the injured CNS remain poorly defined.

Furthermore, the temporal and spatial dynamics of Treg activity are not fully mapped. Most studies rely on cross-sectional “snapshots” rather than longitudinal imaging, leaving gaps in our understanding of when Tregs migrate from the meninges to the parenchyma and how their interactions with microglia evolve over the days following injury [205–207].

Additionally, definitive identification and targeted manipulation of regulatory T cells (Tregs) remain significant challenges, particularly in the setting of acute neuroinflammation.

Ambiguity of Surface Markers. Although the CD4⁺CD25⁺FOXP3⁺ phenotype remains the gold standard for murine Treg identification, reliance on CD25 (IL-2Rα) alone is problematic in inflammatory contexts. CD25 is transiently upregulated on activated effector T cells, increasing the risk of misclassification and overestimation of bona fide Treg populations [208].

Limitations of Genetic Targeting Approaches. Much of the current mechanistic insight derives from transgenic models, which introduce important confounders. Cre–loxP–based systems (for example, FOXP3-Cre) may exhibit “leaky” recombination in non-Treg cells that transiently express FOXP3 during activation. In addition, lineage-tracing strategies can label “ex-Tregs” that have lost suppressive function, thereby complicating interpretation of functional outcomes [83, 84, 209, 210].

Confounders in Depletion Models. Studies employing diphtheria toxin receptor (DTR)–based approaches (such as DEREG mice) to ablate Tregs frequently induce profound systemic immune dysregulation. The abrupt loss of immune restraint can trigger a cytokine surge that independently worsens brain injury, making it difficult to distinguish the specific contribution of Treg loss from secondary inflammation-driven toxicity [85, 86].

### The “Mouse-to-Human” Translational Gap

Findings from murine models do not always predict human responses due to fundamental interspecies differences in T cell receptor repertoires, cytokine signaling, and neurovascular anatomy [28].

This species gap is a major reason why therapies that appear robust in inbred, pathogen-free mice often fail in genetically diverse human populations.

### Challenges in Clinical Implementation

Translating Treg-based therapies (whether adoptive transfer or IL-2 expansion) introduces specific clinical risks that must be managed [211]:

- Off-target immunosuppression: By design, Treg therapies dampen immune activation. Systemic expansion risks suppressing necessary antimicrobial or antitumor responses, predisposing patients to opportunistic infections. A central challenge is achieving CNS-specific regulation without compromising systemic immunity [212, 213].
- Treg stability and phenotypic drift: Maintaining the suppressive phenotype after infusion is critical. In inflammatory environments rich in IL-6, expanded Tregs can lose FOXP3 expression and “drift” toward a proinflammatory Th17-like phenotype, potentially exacerbating rather than resolving injury [209, 214].
- Recent evidence suggests that Treg therapy itself may inadvertently promote this Th17 generation by reducing available IL-2 [215]. Strategies to stabilize the *FOXP3* locus via epigenetic modulation are currently under investigation to prevent this conversion [88, 216].
- Delivery and CNS targeting: Efficiently delivering Tregs to the brain remains a hurdle. Following intravenous infusion, significant numbers of Tregs become sequestered in the spleen or lungs rather than crossing the BBB. Novel delivery routes, such as intranasal administration [217, 218] or biomaterial scaffolds implanted at the injury site [219], may offer solutions to increase CNS bioavailability while limiting systemic exposure.
- Patient variability: Clinical responses will likely vary based on age, genetics, and comorbidities. For example, aging is associated with a shift toward proinflammatory phenotypes (“inflammaging”), which may blunt the efficacy of autologous Treg transfer [220]. Furthermore, concurrent medications common in neurocritical care may interfere with IL-2 signaling and Treg survival.

### Long-Term Safety and Monitoring

Unlike traditional pharmacotherapeutics, Tregs are a “living drug” capable of persistence and expansion. This raises unique safety questions regarding long-term phenotypic stability and the potential for off-target effects [134]. Successful translation will require robust pharmacovigilance frameworks and sensitive assays to track infused cells and monitor for delayed adverse events.

## Conclusion and Future Directions

T lymphocytes are central players in acute neuroinflammation, driving both injury and repair. While proinflammatory subsets like Th1 and Th17 cells disrupt the BBB and amplify tissue damage, Tregs help restore balance by dampening inflammation and supporting tissue repair. Preclinical studies across AIS, ICH, TBI, and aSAH consistently demonstrate that boosting Treg function whether through low-dose IL-2, adoptive transfer, or chemokine-guided recruitment can significantly improve neurological outcomes. However, the path to clinical application requires precision; therapeutic success depends on temporally optimized interventions that support the resolution phase without impeding early, beneficial innate immune responses. Future translational efforts must prioritize early-phase clinical trials to define optimal dosing and safety profiles, alongside the identification of biomarkers, such as Treg:Teff ratios, to predict therapeutic responsiveness. Additionally, developing advanced delivery platforms, including intranasal biologics or nanoparticle-based carriers, will be critical for enhancing CNS specificity and minimizing off-target effects. Ultimately, transitioning from broad immunosuppression to targeted Treg modulation holds significant promise for transforming the management of acute neuroinflammatory diseases.

## Supplementary Information

Below is the link to the electronic supplementary material.

ESM 1 (DOCX 66.1 KB)

## Data Availability

No datasets were generated or analysed during the current study.
