# Supplementary material for: Harnessing Regulatory T Cells to Modulate Acute Brain Injury: From Mechanisms to Therapy
Source: Mol Neurobiol. 2026 Jun 29;63(1):731. doi: 10.1007/s12035-026-06011-7 (PMC13310834; doi:10.1007/s12035-026-06011-7)
Supplement: Supplementary file 1 — (DOCX 66.1 KB) [file 12035_2026_6011_MOESM1_ESM.docx]

**Response to the reviewer**

**Reviewer comments:** The authors have made a meaningful effort to address the major concerns raised in the previous round. In particular, the revised manuscript now includes discussion of Th2 cells, expands the description of Treg-mediated modulation of Th1/Th17 responses and Th2-associated polarization, and provides additional mechanistic depth regarding CTLA-4, NRP1, CD25, infectious tolerance, and non-FOXP3 regulatory subsets. The translational section is also improved by acknowledging key limitations of current Treg-based strategies, including issues related to adoptive transfer, phenotypic stability, delivery, and off-target immunosuppression. Overall, the manuscript is substantially improved compared with the previous version. That said, I still think the manuscript would benefit from one final round of refinement before acceptance. First, although the review is improved, it could still be more tightly focused on its central Treg-centered theme. In several places, the discussion remains somewhat broader than necessary, and the balance between general T-cell biology and the specific role of Tregs in acute brain injury could be further optimized. Second, while the bibliography has clearly been revised, the authors should perform one final careful check to ensure that mechanistic statements are consistently supported by the most context-appropriate primary literature, especially when conclusions are presented in an ABI-specific framework. These remaining issues are relatively minor and appear addressable without major restructuring. For these reasons, my recommendation is minor revision. The manuscript has improved sufficiently in scope, mechanistic depth, and translational relevance, but it would benefit from one final polishing round to sharpen focus and ensure citation precision throughout.

1. *We thank the reviewer for all the comments. First, we have revised the “Key Points” section to better emphasize the central and dynamic role of regulatory T cells (Tregs) in acute neuroinflammation across ischemic stroke, intracerebral hemorrhage, subarachnoid hemorrhage, and traumatic brain injury. We also refined the language to further highlight Treg-mediated modulation of neurovascular inflammation, blood–brain barrier integrity, CNS immune interactions, and emerging translational immunotherapeutic strategies.*

**Updated Version:**

**Key Points:**

1. Regulatory T cells (Tregs) have emerged as central modulators of neuroinflammation across acute brain injuries including ischemic stroke, intracerebral hemorrhage, subarachnoid hemorrhage, and traumatic brain injury.
2. Treg-mediated immune responses evolve dynamically after acute brain injury, transitioning from early peripheral immunomodulation to CNS-resident repair programs that preserve blood–brain barrier integrity and limit secondary neuroinflammation.
3. Tregs regulate interactions between microglia, astrocytes, endothelial cells, and infiltrating leukocytes, thereby promoting neuroprotection and tissue recovery.
4. Emerging therapeutic strategies including low-dose interleukin-2, adoptive Treg transfer, and targeted immune trafficking modulation show promise for improving neurological outcomes after acute CNS injury.
5. Translational approaches targeting Tregs may offer novel immune-directed therapies to mitigate delayed complications including cerebral edema, infarct progression, and vasospasm following severe acute brain injury.
2. *We have updated the CNS immune compartmentalization and glymphatic section, to maintain a stronger Treg-centered focus and reduce broader background discussion.*

Updated section:

**CNS immune compartmentalization and Treg trafficking**

The CNS maintains a specialized form of immune compartmentalization that tightly regulates the trafficking, activation, and function of T lymphocytes, including Tregs, during injury and repair^1^. This immune specialization plays a defining role in acute CNS disorders^2^. At the core of this compartmentalization is the **BBB**, a selectively permeable interface formed by brain microvascular endothelial cells (BMECs), pericytes, astrocytes, and the basement membrane^1,3,4^. Under physiological conditions, the BBB restricts paracellular movement of immune cells and maintains CNS homeostasis^5^. However, in the context of ABI, **BBB disruption** enables the entry of peripheral immune cells including pro-inflammatory Th1 and Th17 cells, as well as Tregs into the parenchyma^6^. Importantly, **Tregs modulate BBB integrity** by downregulating endothelial adhesion molecules such as ICAM-1 and VCAM-1 and secreting anti-inflammatory cytokines like IL-10 and TGF-β, which help restore barrier function and prevent excessive immune infiltration^7,8^.

Within the **neurovascular unit**, Tregs interact with astrocytes and microglia, influencing the inflammatory milieu following acute brain injury^9,10^. Experiment studies in AIS and other CNS injury models suggest that Tregs can modulate glial activation and promote anti-inflammatory or reparative phenotypes within the injured brain^7,11^. Chemokine signaling within the neurovascular unit, including astrocyte-derived mediators that regulate leukocyte trafficking across the BBB, plays an important role in coordinating immune cell recruitment to sites of injury^12,13^.

In this context, Tregs have been shown to attenuate microglial production of pro-inflammatory cytokines such as IL-1β and TNF-α, thereby limiting secondary neuroinflammation and downstream leukocyte recruitment^9,14^. Specifically, Tregs utilize mediators like **interleukin-10 (IL-10)** and **amphiregulin (AREG)** to suppress neurotoxic astrogliosis and promote a shift toward a neuroprotective microglial state^7,11^. Together, these interactions highlight the role of Tregs as modulators of glial responses within the injured CNS microenvironment^15^.

Beyond the BBB, CNS immune regulation is also influenced by glymphatic and meningeal lymphatic drainage pathways, which facilitate clearance of inflammatory mediators and trafficking of CNS-derived antigens toward cervical lymph nodes^16-18^. Impairment of these systems has been observed in AIS and TBI and may contribute to persistent neuroinflammation^16,19^. Emerging evidence suggests that Treg-mediated suppression of astrocytic and inflammatory signaling pathways may indirectly influence glymphatic function, although direct mechanistic links remain incompletely understood^20,21^.

*3. We additionally incorporated a new subsection on Treg plasticity and functional instability in acute neuroinflammation to further strengthen the mechanistic framework of the review. This section highlights the emerging concept that Tregs exhibit context-dependent phenotypic and functional heterogeneity within inflammatory CNS environments, with potential implications for immune regulation and future Treg-directed therapeutic strategies in acute brain injury.*

**New Section:**

**1.2 Treg Plasticity and Functional Instability in Acute Neuroinflammation**

Although Tregs are classically defined by stable FOXP3 expression and immunosuppressive function, accumulating evidence suggests that Tregs exhibit considerable phenotypic plasticity in inflammatory environments^67,82,83^. Pro-inflammatory cytokines including IL-6, IL-1β, TNF-α, and IL-23 can impair Treg stability and promote acquisition of Th1- or Th17-like features through induction of transcription factors such as T-bet and RORγt^83-85^. In certain contexts, destabilized Tregs may produce pro-inflammatory mediators including IL-17A and IFN-γ, although the significance of these transitions in acute CNS injury remains incompletely understood^83,86^.

This concept may be particularly relevant in acute brain injury, where ischemia, DAMP release, oxidative stress, and BBB disruption generate highly inflammatory microenvironments. Experimental studies in AIS and TBI suggest that these conditions may influence Treg suppressive capacity and alter the balance between protective immune regulation and persistent neuroinflammation^18,27,87^. At the same time, emerging evidence indicates that CNS-associated Tregs can acquire tissue-adapted reparative phenotypes involved in oligodendrogenesis, microglial modulation, and tissue remodeling through mediators such as amphiregulin, IL-10, TGF-β, and CCN3^61,75,76^.

Recognition of Treg heterogeneity and functional plasticity has important therapeutic implications. Strategies such as low-dose IL-2 or adoptive Treg transfer may ultimately require not only expansion of Treg populations, but also stabilization of their regulatory phenotype within the inflammatory CNS microenvironment^67,88^.

4. *We revised the therapeutic strategies section to improve organization and reduce redundancy. Specifically, we removed the standalone “Broader Therapeutic Implications” subsection and consolidated relevant content into the preceding therapeutic sections. This revision streamlined the narrative, eliminated repetitive discussion of matrix metalloproteinase (MMP)-mediated mechanisms, and improved the overall flow of the therapeutic framework while preserving all key scientific and translational concepts.*

*5. We carefully reviewed and updated Table 2 to improve accuracy and consistency with the revised reference list. Several citations were corrected following manuscript reorganization and reference renumbering, and supporting references were refined to better align with the biological functions and neuroinflammatory significance of the markers discussed. These revisions improve the accuracy and clarity of the table while ensuring that each marker is supported by the most relevant literature.*

1.	Carson MJ, Doose JM, Melchior B, Schmid CD, Ploix CC. CNS immune privilege: hiding in plain sight. Immunol Rev 2006;213:48-65. (In eng). DOI: 10.1111/j.1600-065X.2006.00441.x.

2.	Cervantes-Barragán L, Firner S, Bechmann I, et al. Regulatory T cells selectively preserve immune privilege of self-antigens during viral central nervous system infection. J Immunol 2012;188(8):3678-85. (In eng). DOI: 10.4049/jimmunol.1102422.

3.	Daneman R, Prat A. The blood-brain barrier. Cold Spring Harb Perspect Biol 2015;7(1):a020412. (In eng). DOI: 10.1101/cshperspect.a020412.

4.	Raposo C, Graubardt N, Cohen M, et al. CNS repair requires both effector and regulatory T cells with distinct temporal and spatial profiles. J Neurosci 2014;34(31):10141-55. (In eng). DOI: 10.1523/jneurosci.0076-14.2014.

5.	Engelhardt B, Ransohoff RM. Capture, crawl, cross: the T cell code to breach the blood-brain barriers. Trends Immunol 2012;33(12):579-89. (In eng). DOI: 10.1016/j.it.2012.07.004.

6.	Iadecola C, Anrather J. The immunology of stroke: from mechanisms to translation. Nat Med 2011;17(7):796-808. (In eng). DOI: 10.1038/nm.2399.

7.	Liesz A, Suri-Payer E, Veltkamp C, et al. Regulatory T cells are key cerebroprotective immunomodulators in acute experimental stroke. Nat Med 2009;15(2):192-9. (In eng). DOI: 10.1038/nm.1927.

8.	Li P, Gan Y, Sun BL, et al. Adoptive regulatory T-cell therapy protects against cerebral ischemia. Ann Neurol 2013;74(3):458-71. (In eng). DOI: 10.1002/ana.23815.

9.	Xie L, Choudhury GR, Winters A, Yang SH, Jin K. Cerebral regulatory T cells restrain microglia/macrophage-mediated inflammatory responses via IL-10. Eur J Immunol 2015;45(1):180-91. (In eng). DOI: 10.1002/eji.201444823.

10.	Zheng Y, Ren Z, Liu Y, et al. T cell interactions with microglia in immune-inflammatory processes of ischemic stroke. Neural Regen Res 2025;20(5):1277-1292. (In eng). DOI: 10.4103/nrr.Nrr-d-23-01385.

11.	Choi J, Kim BR, Akuzum B, Chang L, Lee JY, Kwon HK. T(REG)king From Gut to Brain: The Control of Regulatory T Cells Along the Gut-Brain Axis. Front Immunol 2022;13:916066. (In eng). DOI: 10.3389/fimmu.2022.916066.

12.	Yang C, Hawkins KE, Doré S, Candelario-Jalil E. Neuroinflammatory mechanisms of blood-brain barrier damage in ischemic stroke. American Journal of Physiology-Cell Physiology 2019;316(2):C135-C153.

13.	Wang Y, Liu W, Geng P, et al. Role of Crosstalk between Glial Cells and Immune Cells in Blood-Brain Barrier Damage and Protection after Acute Ischemic Stroke. Aging Dis 2023;15(6):2507-2525. (In eng). DOI: 10.14336/ad.2023.1010.

14.	Benakis C, Simats A, Tritschler S, et al. T cells modulate the microglial response to brain ischemia. Elife 2022;11 (In eng). DOI: 10.7554/eLife.82031.

15.	Harkins AL, Kopec AL, Keeler AM. Regulatory T Cell Therapeutics for Neuroinflammatory Disorders. Crit Rev Immunol 2022;42(2):1-27. (In eng). DOI: 10.1615/CritRevImmunol.2022045080.

16.	Iliff JJ, Chen MJ, Plog BA, et al. Impairment of glymphatic pathway function promotes tau pathology after traumatic brain injury. J Neurosci 2014;34(49):16180-93. (In eng). DOI: 10.1523/jneurosci.3020-14.2014.

17.	Louveau A, Smirnov I, Keyes TJ, et al. Structural and functional features of central nervous system lymphatic vessels. Nature 2015;523(7560):337-341.

18.	Rustenhoven J, Drieu A, Mamuladze T, et al. Functional characterization of the dural sinuses as a neuroimmune interface. Cell 2021;184(4):1000-1016.e27. (In eng). DOI: 10.1016/j.cell.2020.12.040.

19.	Gaberel T, Gakuba C, Goulay R, et al. Impaired glymphatic perfusion after strokes revealed by contrast-enhanced MRI: a new target for fibrinolysis? Stroke 2014;45(10):3092-6. (In eng). DOI: 10.1161/strokeaha.114.006617.

20.	Krämer TJ, Hack N, Brühl TJ, et al. Depletion of regulatory T cells increases T cell brain infiltration, reactive astrogliosis, and interferon-γ gene expression in acute experimental traumatic brain injury. J Neuroinflammation 2019;16(1):163. (In eng). DOI: 10.1186/s12974-019-1550-0.

21.	Ito M, Komai K, Mise-Omata S, et al. Brain regulatory T cells suppress astrogliosis and potentiate neurological recovery. Nature 2019;565(7738):246-250.
